# Supplementary material for: Chromosome-level baobab genome illuminates its evolutionary trajectory and environmental adaptation
Source: Nat Commun. 2024 Oct 12;15:8833. doi: 10.1038/s41467-024-53157-w (PMC11470940; doi:10.1038/s41467-024-53157-w)
Supplement: Supplementary file 1 — Supplementary Information [file 41467_2024_53157_MOESM1_ESM.pdf]

Chromosome-level baobab genome illuminates its evolutionary trajectory and environmental adaptation

Kitony *et al.*

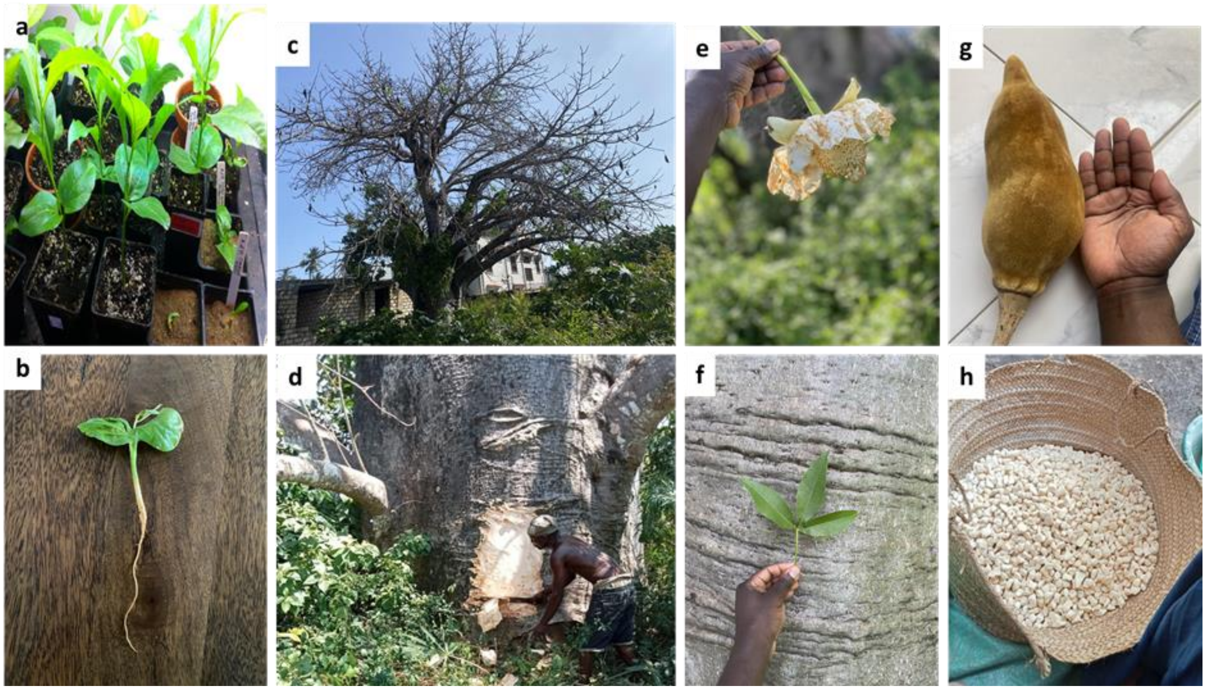

Supplementary Fig. 1. Baobab (*Adansonia digitata*) is an economically important tree. a USDA-GRIN PI 77271 seedlings used for genome sequencing. **b** Seedling tap root. **c** Deciduous mature tree in its natural habitat: a representation of a long history of coexistence with humans. **d** Bark harvesting for fiber production in Kwale, Kenya. **e** Whitish waxy flower that has a diameter of up to 20 cm (8"). **f** Shiny reflective grayish surface of mature bark and juvenile leaf. **g** Yellowish hard woody pod of mature fruit with lengths of up to 30 cm (12"). **h** Powdery**,** whitish fruit pulp, which is abundant in vitamin C, antioxidants, calcium, potassium, and dietary fiber.

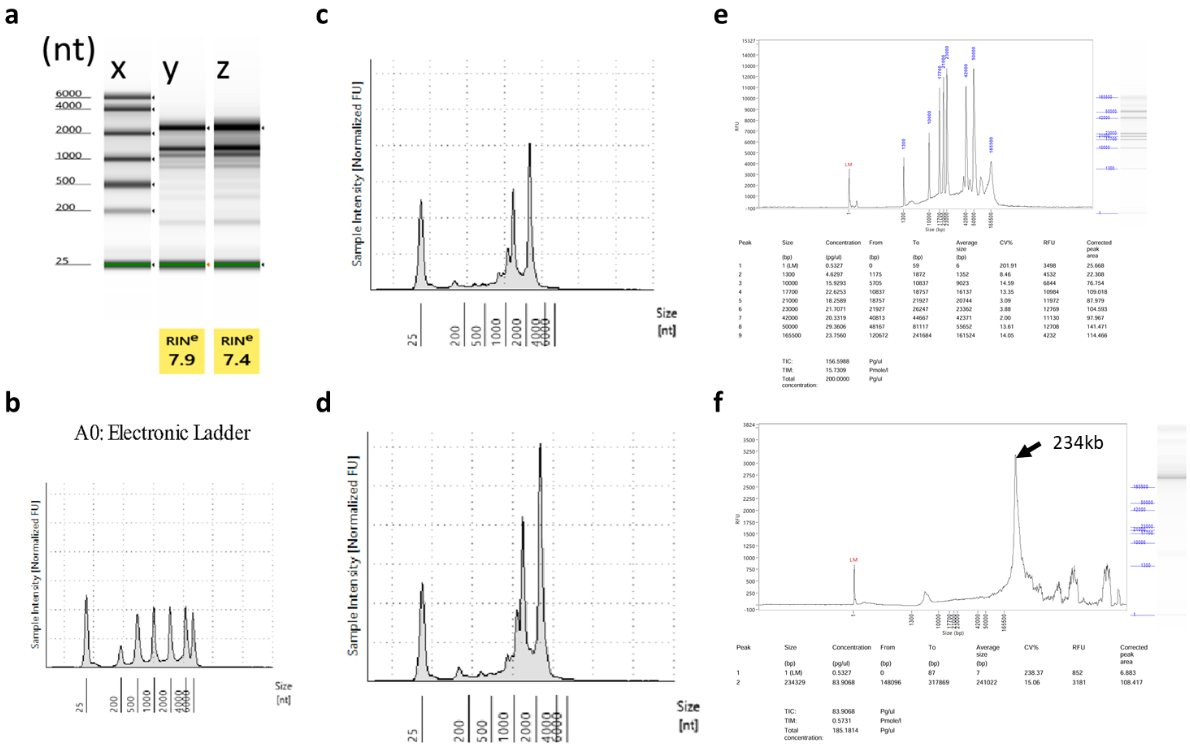

Supplementary Fig. 2. RNA and DNA quality summary. a Gel image and RNA Integrity Number (RIN) values for Ad77271a and Ad77271b RNA; x corresponds to the electronic ladder, y is Ad77271a, and z is Ad77271b. **b** RNA electronic ladder. **c** Electropherograms for Ad77271a. **d** Electropherograms for Ad77271b. **e** DNA electronic ladder. **f** Baobab DNA run on Femto pulse, majority of the DNA was over 165kb in length with main peak estimated at 234kb.

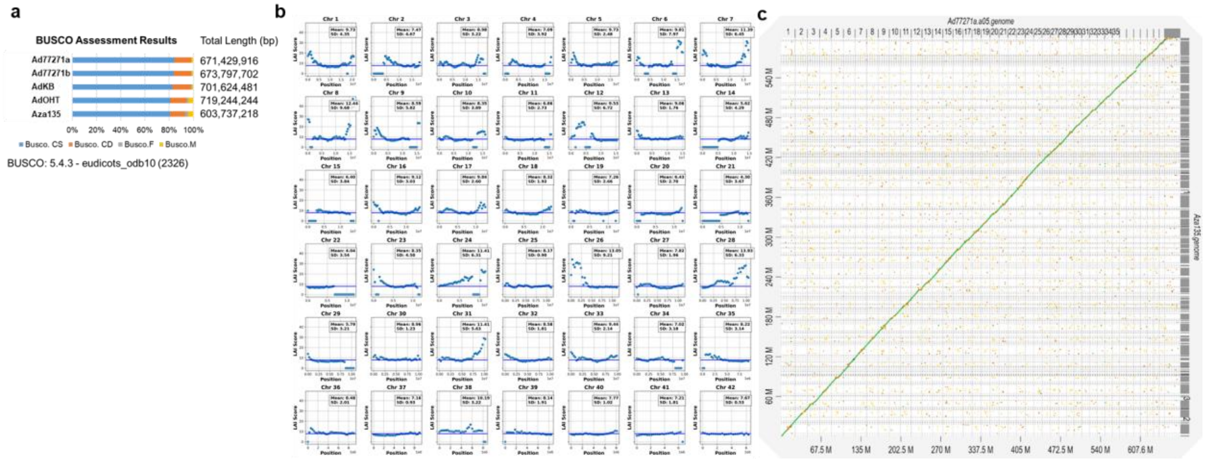

Supplementary Fig. 3. Evaluation of baobab genome assemblies. a Benchmarking Universal Single-Copy Orthologs (BUSCO) assessment showing the completeness of Ad77271a, Ad77271b, AdKB, AdOHT and Aza135 baobab genomes. **b** LTR Assembly Index (LAI) for Ad77271a chromosomes. The x-axis displays the position along the chromosomes, while the dots represent LAI scores calculated using 3 Mb sliding windows with 300 kb steps after running LTR_retriever (https://github.com/oushujun/LTR_retriever). The blue line denotes the average LAI score for each chromosome. **c** Dot plot of Ad77271a against a scaffolded Aza135 genome.

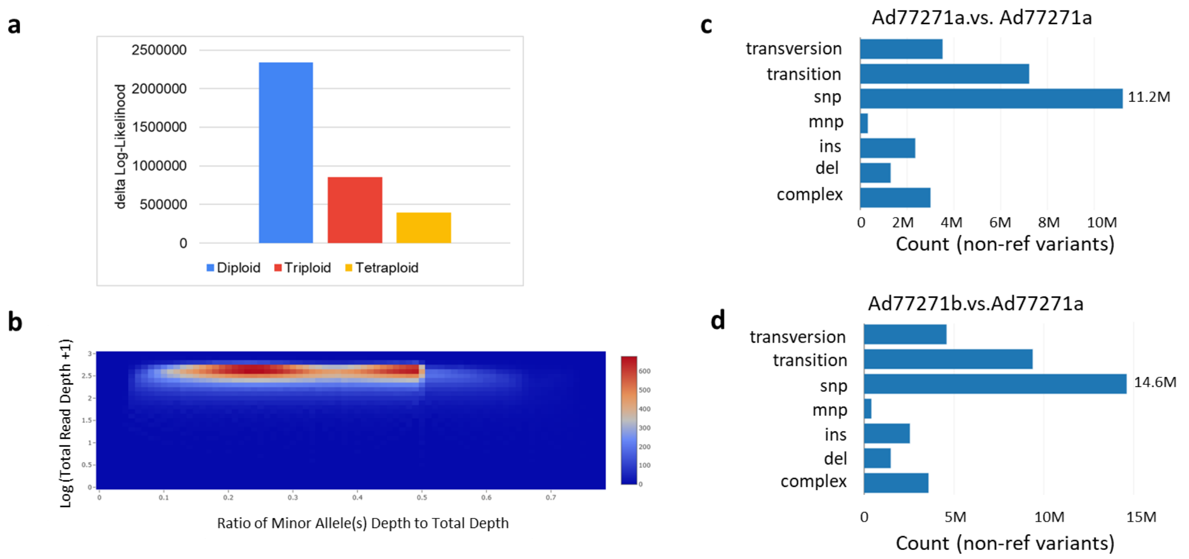

Supplementary Fig. 4. Variant analysis reveals autotetraploidy in the *Adansonia digitata* genome. a Gaussian Mixture Model (GMM) estimation of ploidy. This models frequency distributions at variant sites with two segregating bases and uses maximum likelihood to pick the most likely model[1]. The ploidy level with the smallest Δ logL is identified as the true ploidy (tetraploid for baobab). **b** Two-dimensional histogram illustrates ploidy based on minor allele frequency coverage for Ad77271b; the sibling genome of Ad77271a. For diploid organisms, a single peak is expected. However, for tetraploid organisms, the histogram should exhibit two peaks, approximately located at 0.25 and 0.5. The summary of different variants is shown for **c** Ad77271a vs. Ad77271a **d** Ad77271b vs. Ad77271a. For diploid loci and homozygous alternates, it would contribute two points. For heterozygous, it would contribute one point.

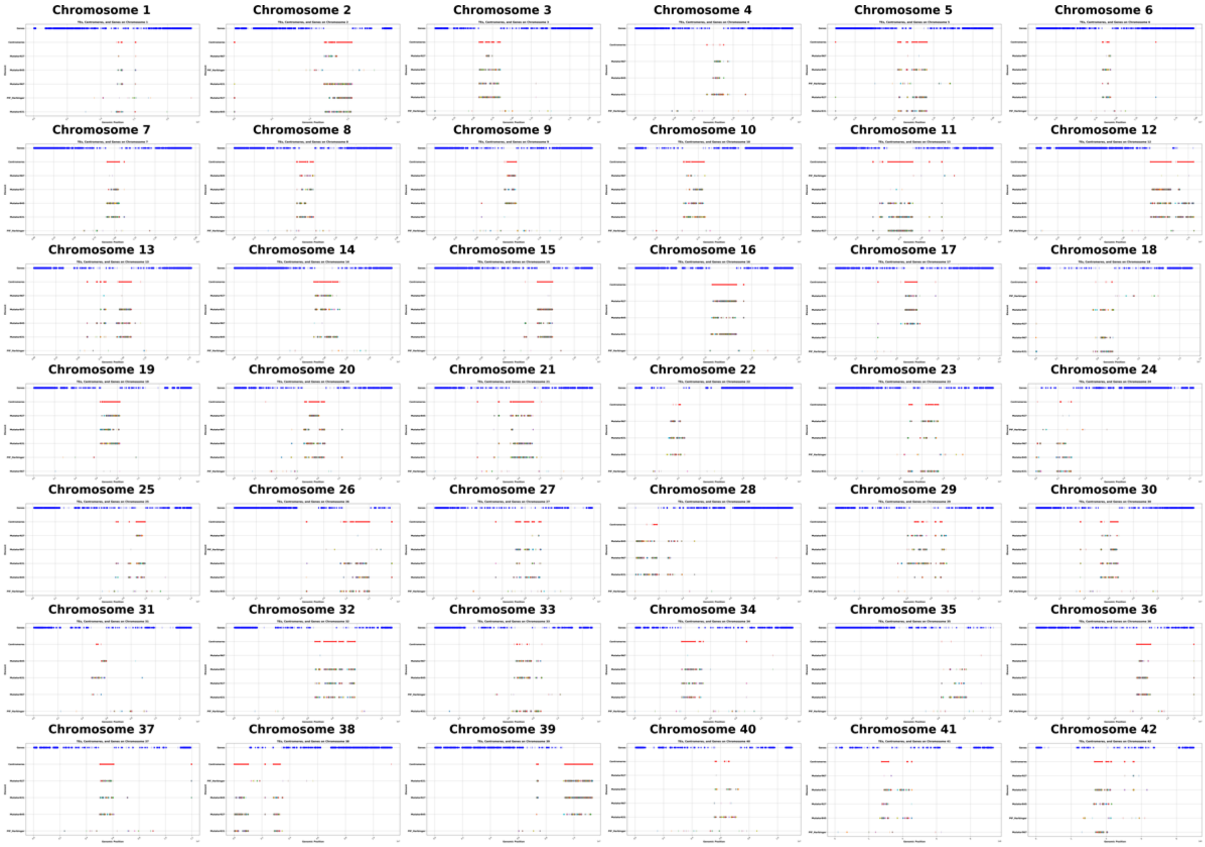

Supplementary Fig. 5. Global plots of the relationship between DNA mutators and centromeres in Ad77271a. Four mutator Terminal Inverted Repeat (TIR) transposons (TE_00000631: Mutator631, TE_00000927: Mutator927, TE_00000845: Mutator845, and TE_00000967: Mutator967) are shown. The accumulation of these mutators indicates their involvement in centromere dynamics.

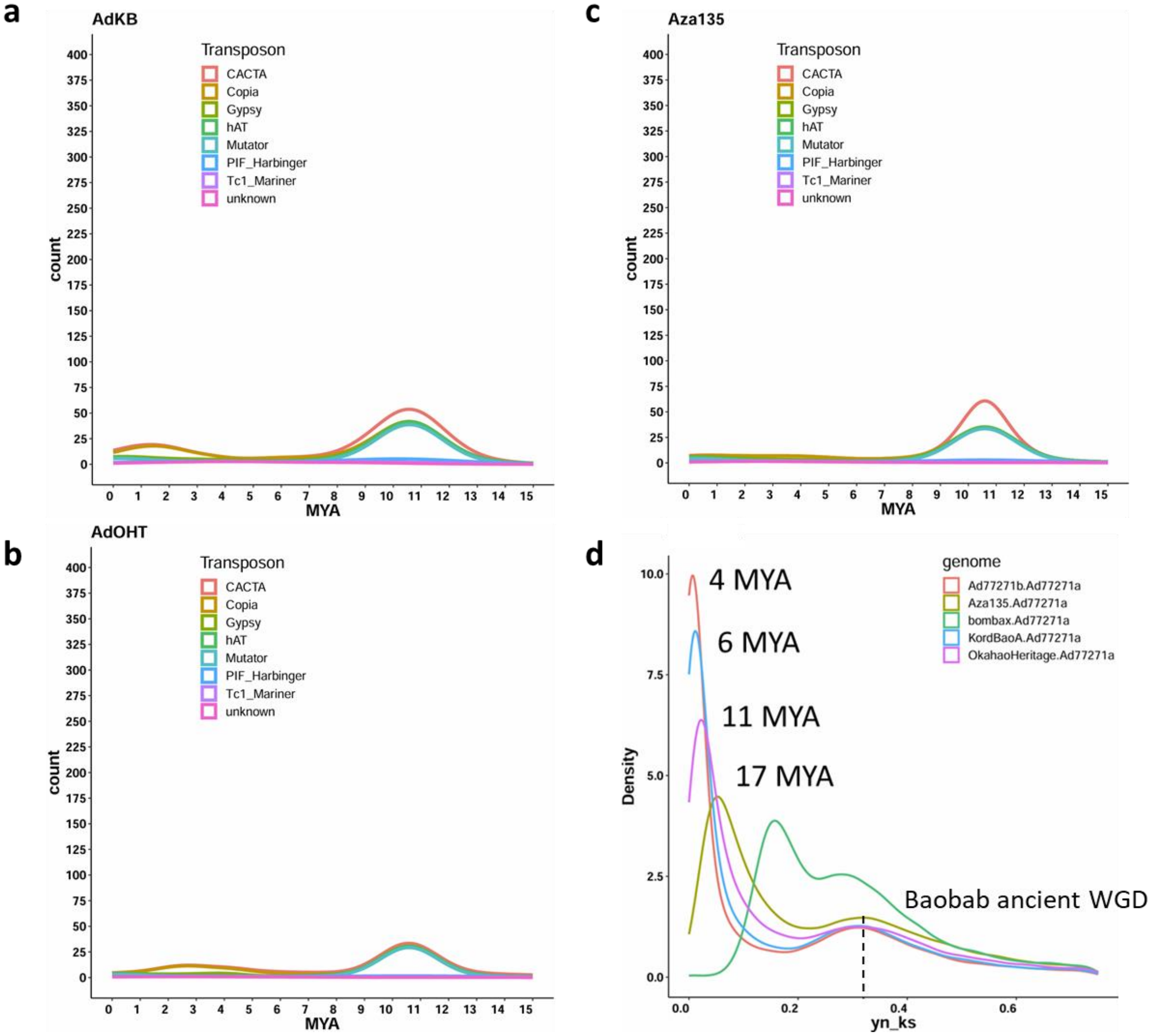

Supplementary Fig. 6. Transposable elements (TEs) evolution and baobab whole genome multiplication (WGM) events. (**a**, **b**, and **c**) Density plots for AdKB, AdOHT, and Aza135 TEs bursts relative to estimated insertion periods in Million Years Ago (MYA). Around 10-12 MYA, the baobab genome experienced elevated levels of CACTA and COPIA long terminal repeat retrotransposons (LTR-RTs). Additionally, TEs proliferated around 3-4 MYA. **d** Ks (synonymous substitution rate) distribution in baobab. The distance between the Ad77271a and Ad77271b siblings likely represents the time of autotetraploidization, as they represent distinct and random haplotypes. The peaks at 4, 6, 11, and 17 MYA for Ad77271b, AdKB, AdOHT, and Aza135 regions indicate baobab duplication or accession split events. Source data are provided as a Source Data file.

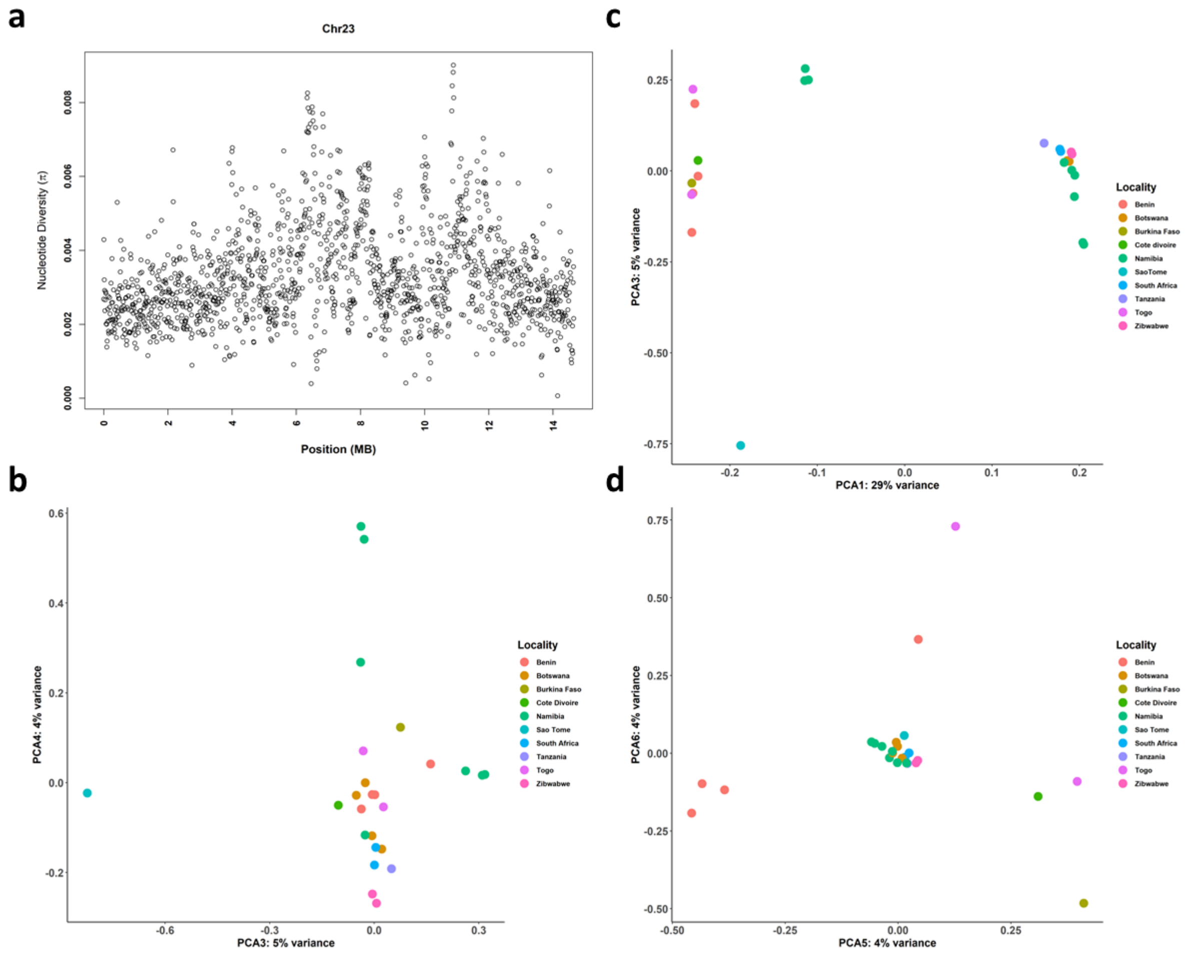

Supplementary Fig. 7. Genetic diversity and population structure of African baobab. a Nucleotide diversity along chromosome 23 in 25 African baobab populations. Chromosome 23 harbored a translocation distinguishing *A. digitata* from *A. za* species (Fig. 1e). Principal Component Analysis (PCA) colored by locality. Panels represent clustering using 6490 SNPs in 25 Adansonia populations: **b** axis 3 vs axis 4; **c** axis 1 vs axis 3; **d** axis 5 vs axis 6.

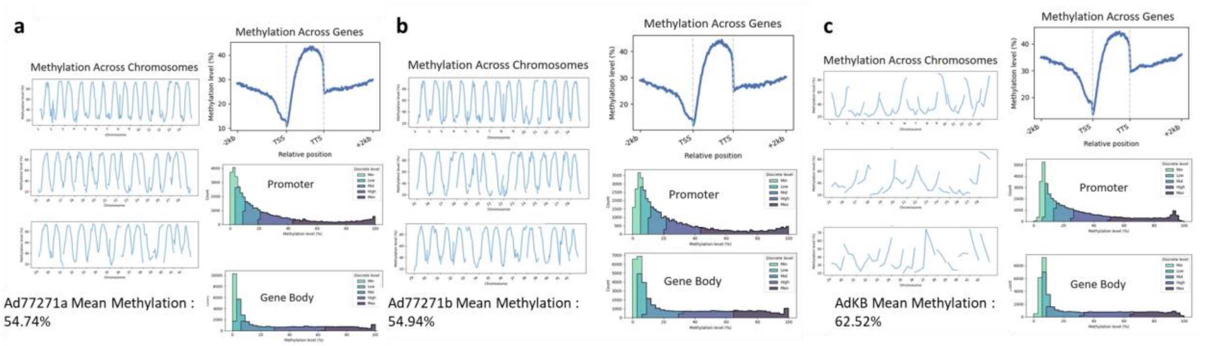

Supplementary Fig. 8. Assessment of methylation levels in baobab genomes. Analysis of methylation across the 42 chromosomes (first column) and mean percentage methylation; methylation patterns across genes, promoters, and gene bodies (second column) for genomes: **a** Ad77271a, **b** Ad77271b, and **c** AdKB.

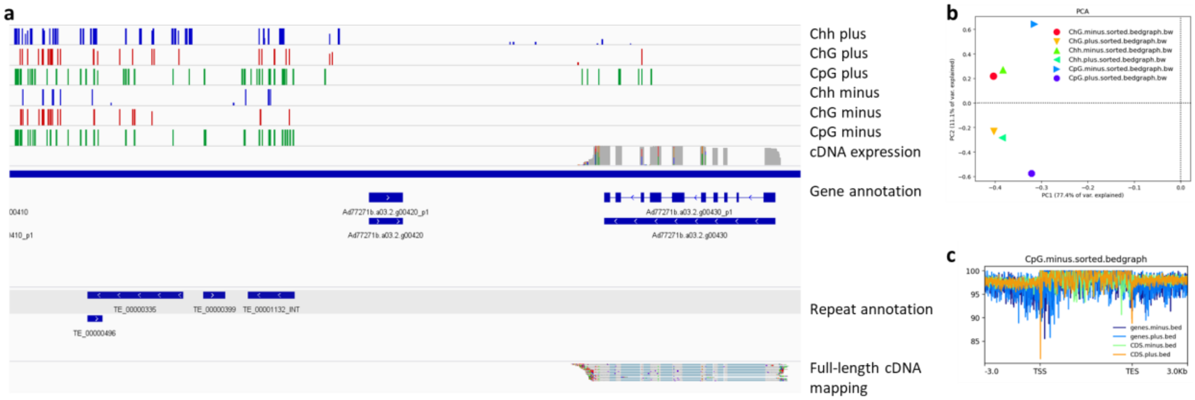

Supplementary Fig. 9. Unveiling subtle methylation patterns in the baobab genome. a Hypermethylation in transposable elements contrasted with hypomethylation of genes along chromosome 2 of Ad77271b. **b** Correlation of methylation on the same strand with varied 5mC methylation types; and **c** Enhanced methylation in gene bodies and specific coding regions compared to intergenic regions.

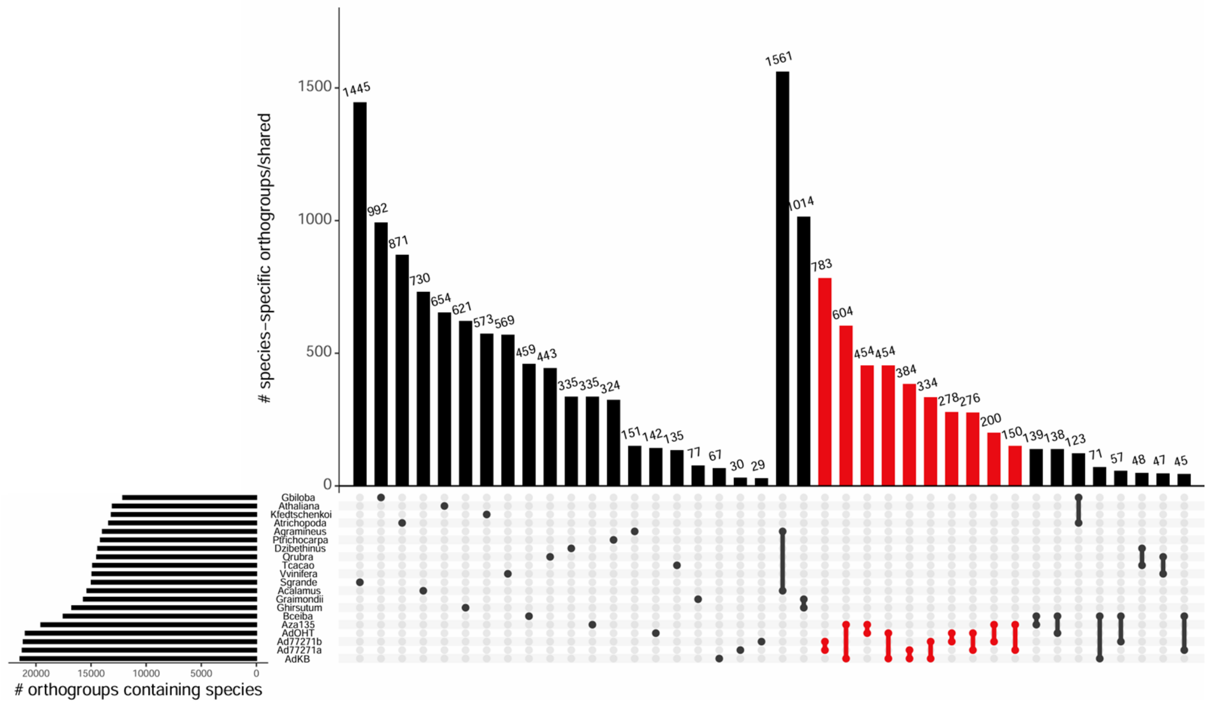

Supplementary Fig. 10. An UpSet plot showing orthogroups across 20 plant species. The 20 plant species inlcude *A. digitata* (Ad77271a, Ad77271b, AdKB,AdOHT), *A. za* (Aza135), *A. calamus*, *A. gramineus*, *A.thaliana*, *A trichopoda*, *B. ceiba*, *D. zibethinus*, *G. biloba*, *G. hirsutum*, *G. raimondii*, *K. fedtschenkoi*, *P. trichocarpa*, *Q. rubra*, *S grande*, *T. cacao*, and *V. vinifera*. The number of species-specific orthogroups and shared orthogroups is shown on the main bar y-axis; red bars correspond to the baobabs. The x-axis bars correspond to the number of orthogroups containing species. Orthologs were characterized using OrthoFinder (v2.5.5) [2].

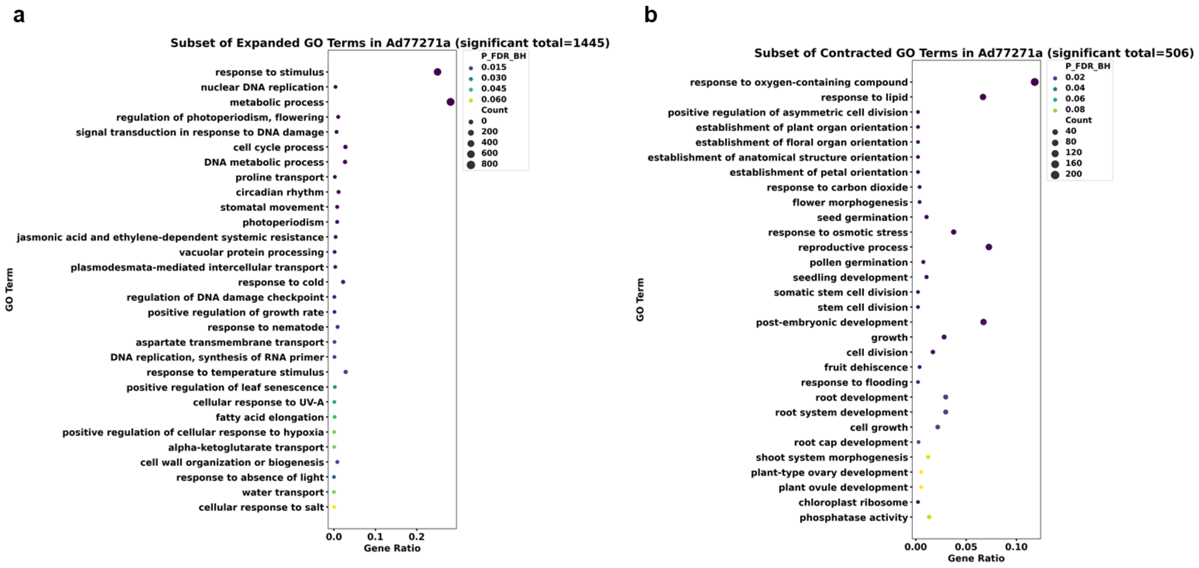

Supplementary Fig. 11. Biological process genes expanded during baobab evolution. a GO enrichment analysis of a subset of 1,145 significantly expanded gene families in Ad77271a. Notable enriched GO terms include cold response (GO:0009409), signal transduction in response to DNA damage (GO:0042770), and circadian rhythm (GO:0007623), highlighting key biological processes that contribute to the long-term survival of baobab. Bar chart displaying GO terms significantly enriched in *Adansonia digitata* (Ad77271a) genes. Cluster of terms related to stress response, including response to wounding (GO:0009611), cell death (GO:0008219) and circadian rhythm (GO:007623). **b** A subset of the 506 significantly contracted GO terms, covering biological processes related to plant growth, development, reproduction, cell division, morphogenesis, organ development, homeostasis, and environmental interactions. Statistical analysis was performed using GOATOOLS [3].

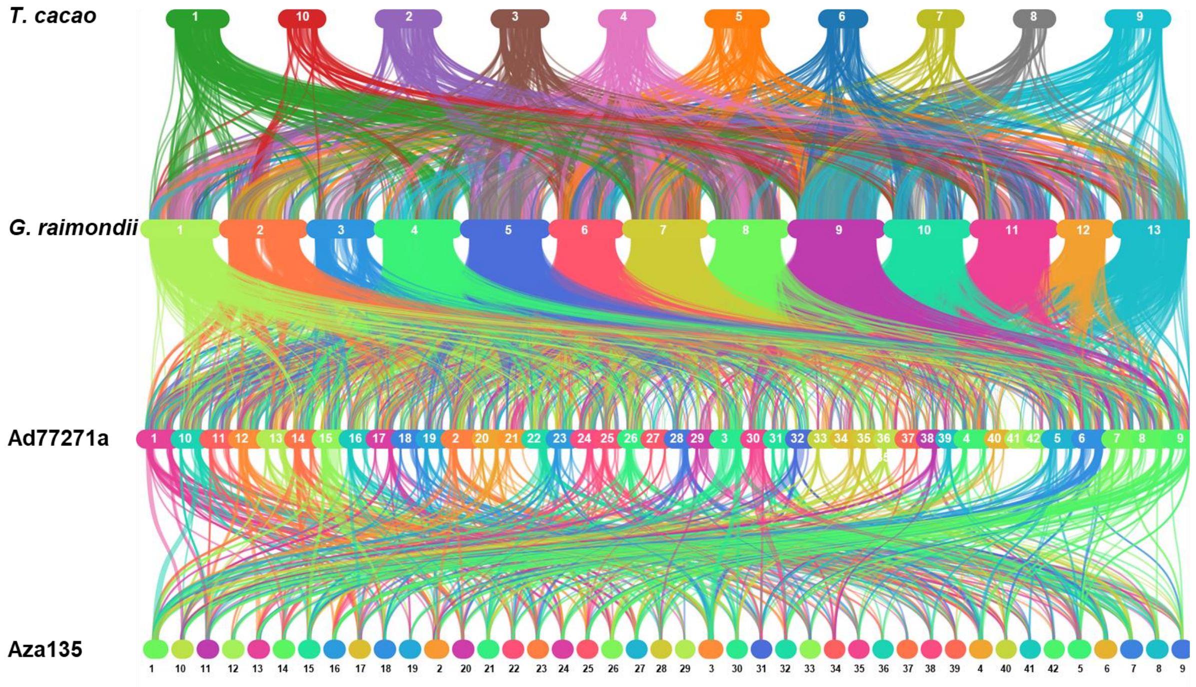

Supplementary Fig. 12. Collinearity between Ad77271a, Aza135, *T. cacao and G. raimondii*. The analysis reveals chromosomal rearrangements. Notably, Aza135 exhibits even smaller chromosomes and a high number, underscoring the extent of chromosomal restructuring after the split in Malvaceae species. The plot was generated using *Theobroma cacao* v2.1 and *Gossypium raimondii* v2.1 annotation from Phytozome v13, the baobab annotations are from the present study; and visualization was done using OrthoVenn3 (OrthoVenn3 (bioinfotoolkits.net).

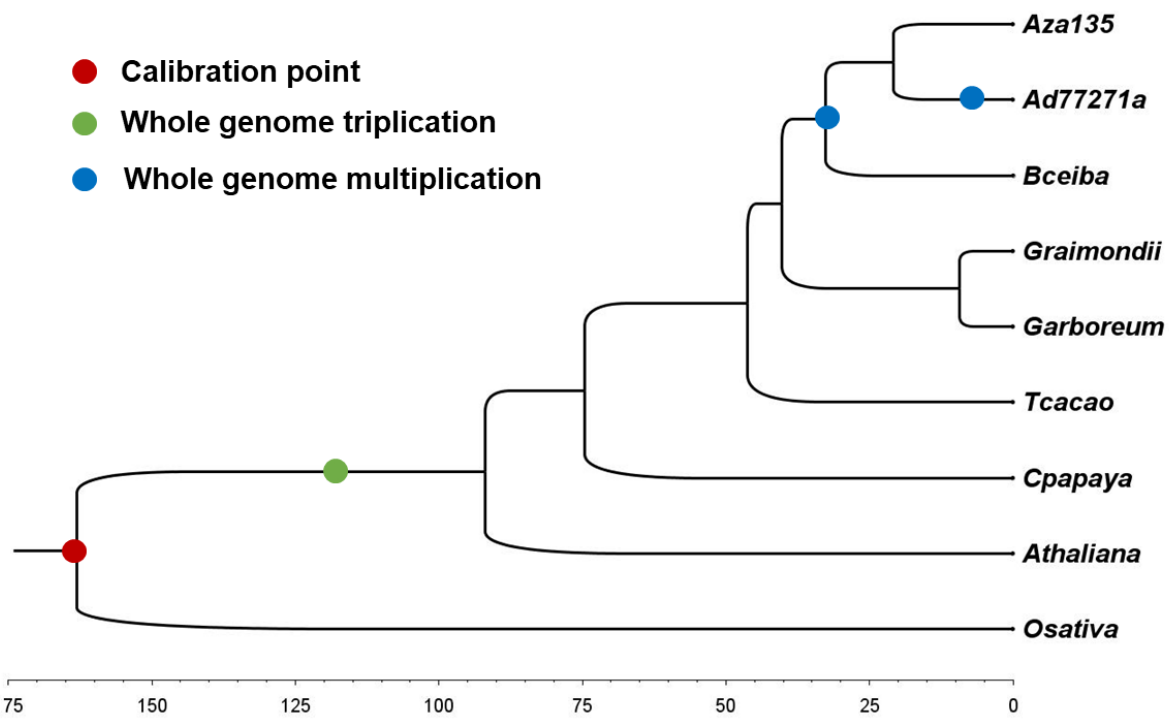

Supplementary Fig. 13. Time-calibrated phylogenetic tree of *Adansonia digitata* (Ad77271a), *Adansonia za* (Aza135*)*, *Bombax ceiba*, *Gossypium raimondii*, *Gossypium arboreum*, *Theobroma cacao*, *Carica papaya*, *Arabidopsis thaliana*, and *Oryza sativa*. The phylogenetic analysis is based on the protein sequences for the 9 plants with *O. sativa* as an outgroup. The timescale in million years ago (MYA) is provided at the bottom. A. *thaliana* to *O. sativa* (142.1–163.5 MYA) (http://www.timetree.org/) was used for calibration. Source data are provided as a Source Data file.

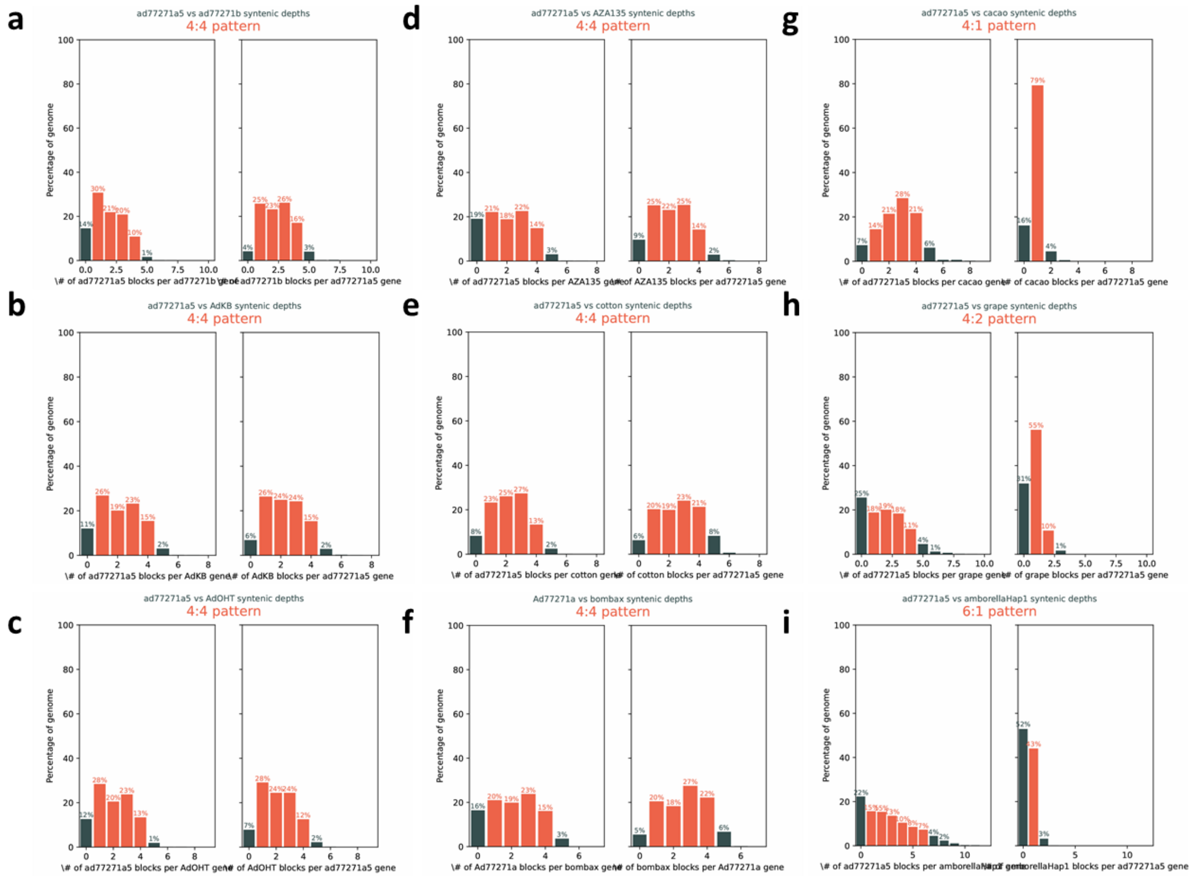

Supplementary Fig. 14. Syntenic depth ratios between Ad77271a and other species. Panels **a** to **i** show syntenic depth ratios between Ad77271a and various species: **a** Ad77271b **b** AdKB **c** AdOHT **d** Aza135 **e** cotton **f** bombax **g** cacao **h** grape and **i** amborella. Source data are provided as a Source Data file

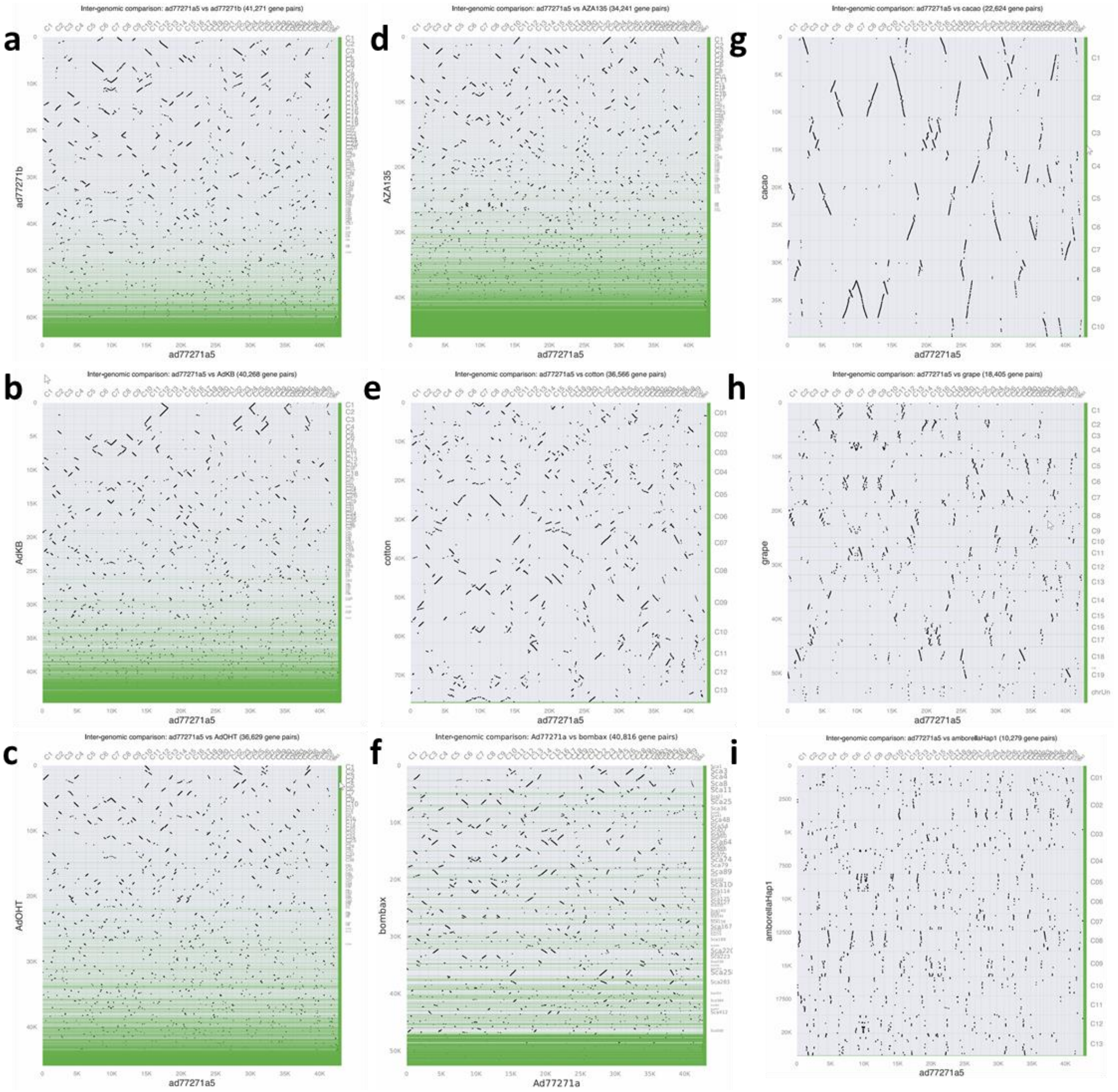

Supplementary Fig. 15. Dot plots between Ad77271a and other species. Panels **a** to **i** show dot plots between Ad77271a and various species: **a** Ad77271b **b** AdKB **c** AdOHT **d** Aza135 **e** cotton **f** bombax **g** cacao **h** grape, and **i** amborella.

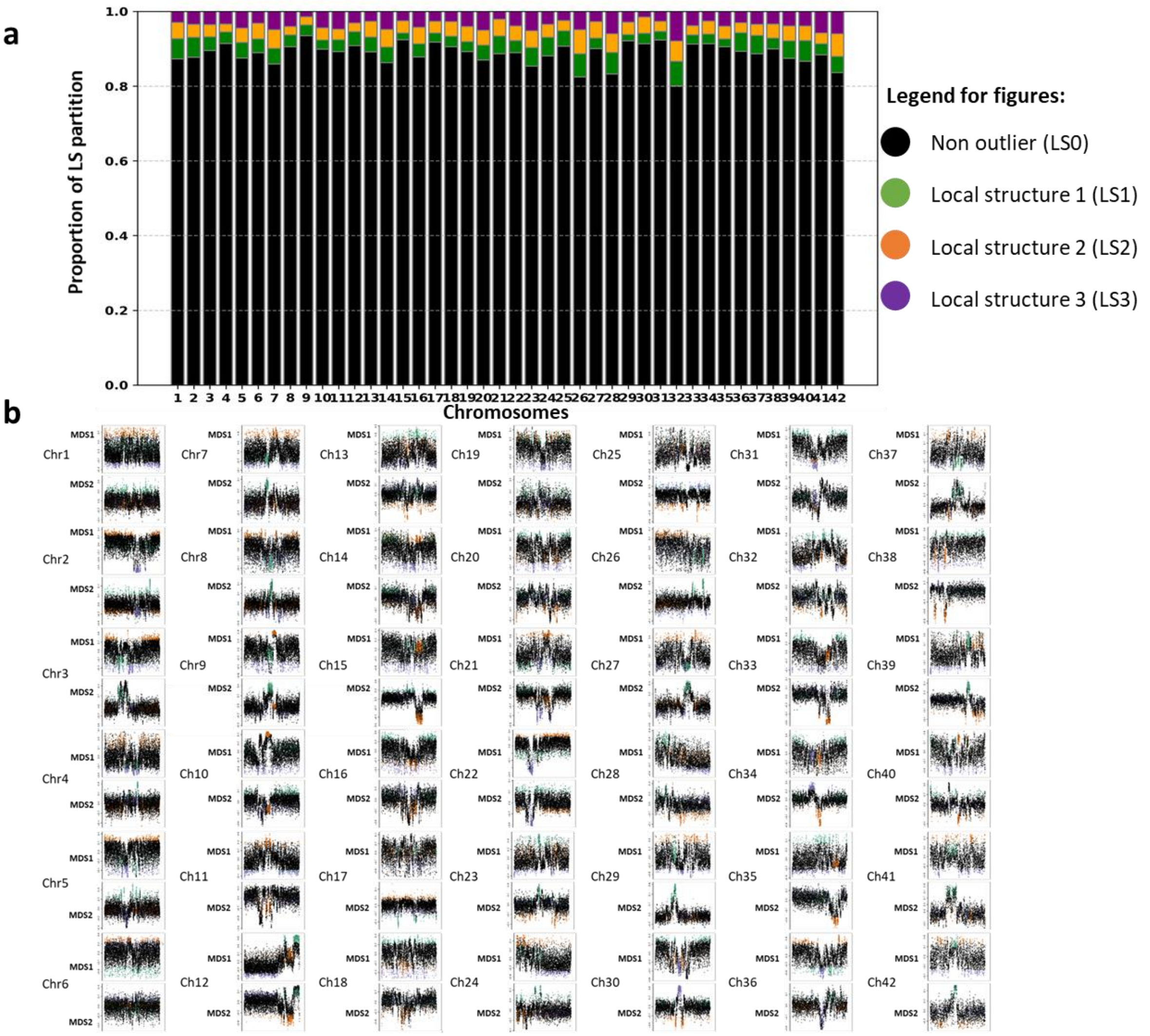

Supplementary Fig. 16. Lostruct partitions vary across *A. digitata* chromosomes. a Proportion of chromosomes assigned to LS0 (black), LS1 (green), LS2 (orange), LS3 (purple) in lostruct partitions. Lostruct uses principal component analysis and multidimensional scaling (MSDS) [4] **b** Local population structure analysis revealed outlier subsets on the 42 chromosomes. We identified three distinct outlier subsets, labeled LS1 (green), LS2 (orange), and LS3 (purple). These subsets were then compared against the rest of the genome, which represents the non-outliers (black) on 3kb windows. Chromosome sizes are not to scale. Source data are provided as a Source Data file.

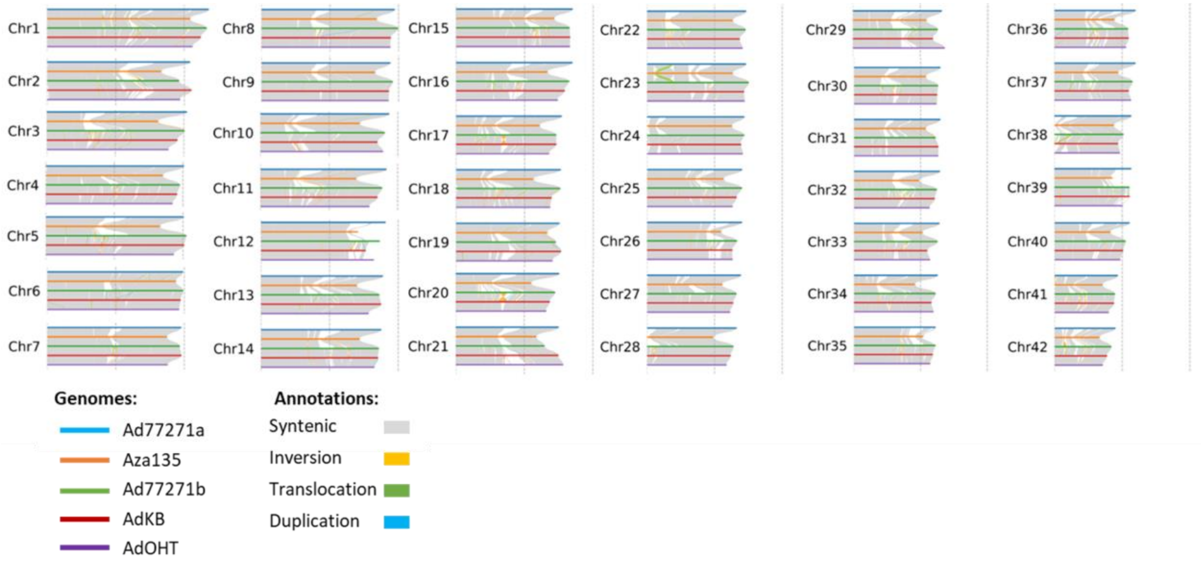

Supplementary Fig. 17. Structural rearrangements and synteny between *A. digitata* (Ad77271a, Ad77271b, AdKB, and AdOHT) and *A. za* (Aza135). Gray, orange, green, and blue-ribbon colors represent syntenic, inversion, translocation, and duplication structural variations, respectively. Structural variations (SVs) were profiled using SyRI v1.6.3 [5].

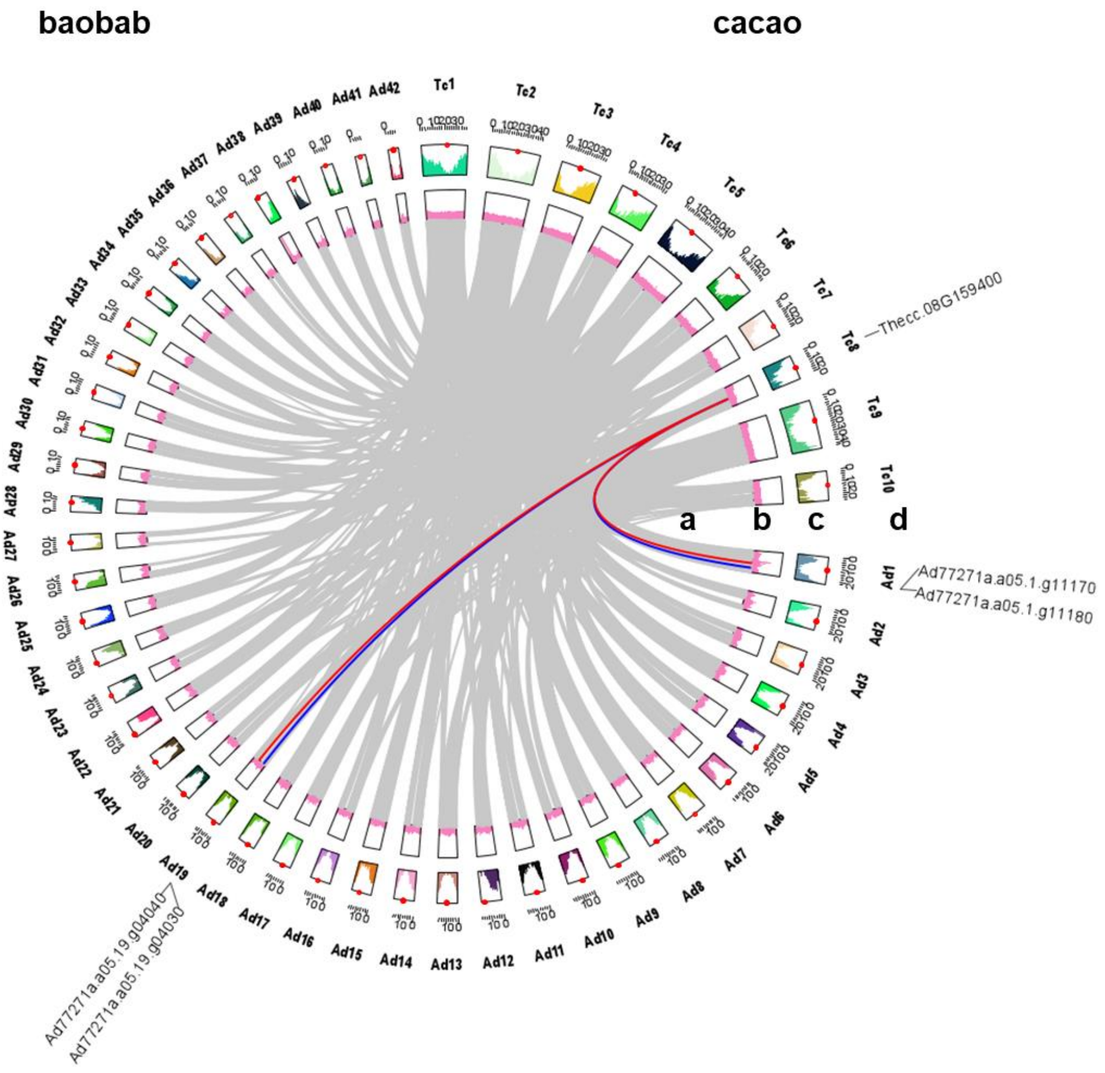

Supplementary Fig. 18. Comparative analysis of autotetraploid *A. digitata* and diploid *Theobroma cacao* genomes. Inner to outer tracks depict: **a** Syntenic genes, **b** GC content, **c** Gene density, and **d** Chromosome information. Prefixes 'Ad' and 'Tc' denote baobab and cacao respectively. The circos plot illustrates 42 pseudomolecules for baobab and 10 for cacao, with a window size of 100 kb. The red asterisk highlights the metacentric and acrocentric centromeres in baobab.

Supplementary Table 1. Summary for 18s, 5.8s, 26s, and 5s rDNA in baobab genomes.

Genome	18s (#)	5.8s (#)	26s (#)	5s (#)
Ad77271a	104	100	75	465
Ad77271b	92	87	61	497
AdKb	54	10	48	208
AdOHT	92	61	87	44
Aza135	54	20	46	67

Supplementary Table 2. Summary of orthogroups and species-unique genes, with protein sequence source shown.

#	Plant	Orthogroups	Specific genes	Reference (Phytozome or links)
1	*Arabidopsis thaliana*	722	3531	*Arabidopsis thaliana* Araport11
2	*Kalanchoe fedtschenkoi*	648	2108	*Kalanchoe fedtschenkoi* v1.1
3	*Ginkgo biloba*	1060	4172	https://ginkgo.zju.edu.cn/genome/ftp/ Version-2021
4	*Amborella trichopoda*	890	4567	*Amborella trichopoda* v1.0
5	*Acorus gramineus*	168	529	*Acorus gramineus* genome assembly ASM3073783v1 - NCBI - NLM (nih.gov)
6	*Populus trichocarpa*	373	1567	Populus trichocarpa v4.1
7	*Quercus rubra*	492	2417	*Quercus rubra* v2.1
8	*Durio zibethinus*	412	1587	*Durio zibethinus* genome assembly Duzib1.0 - NCBI - NLM (nih.gov)
9	*Syzygium grande*	1489	6061	*Syzygium grande* genome assembly NTU_Sgrande_1.0 - NCBI - NLM (nih.gov)
10	*Theobroma cacao*	150	511	*Theobroma cacao* v2.1
11	*Vitis vinifera*	641	2204	*Vitis vinifera* v2.1
12	*Acorus calamus*	790	2431	*Acorus calamus* genome assembly ASM3073784v1 - NCBI - NLM (nih.gov)
13	*Gossypium raimondii*	89	245	*Gossypium raimondii* v2.1
14	*Gossypium hirsutum*	685	2190	*Gossypium hirsutum* UGA230 v1.1
15	*Bombax ceiba*	581	2101	http://gigadb.org/dataset/view/id/100445
16	*Ad77271b*	89	239	This study
17	*Ad77271a*	107	283	This study
18	*Carica papaya*	-	-	*Carica papaya* ASGPBv0.4
19	*Gossypium arboreum*	-	-	doi.org/10.1038/s41588-020-0607-4
20	*Oryza sativa*	-	-	*Oryza sativa* v7.0

Supplementary references

1. Weiß, C. L., Pais, M., Cano, L. M., Kamoun, S. & Burbano, H. A. nQuire: a statistical framework for ploidy estimation using next generation sequencing. *BMC Bioinformatics* **19**, 122 (2018).

2. Emms, D. M. & Kelly, S. OrthoFinder: phylogenetic orthology inference for comparative genomics. *Genome Biol.* **20**, 238 (2019).

3. Klopfenstein, D. V. *et al.* GOATOOLS: A Python library for Gene Ontology analyses. *Sci. Rep.* **8**, 10872 (2018).

4. 2. Li, H. & Ralph, P. Local PCA Shows How the Effect of Population Structure Differs Along the Genome. *Genetics* **211**, 289–304 (2019).

5. Goel, M., Sun, H., Jiao, W.-B. & Schneeberger, K. SyRI: finding genomic rearrangements and local sequence differences from whole-genome assemblies. *Genome Biol.* **20**, 277 (2019).
