# Supplementary material for: Chromosome-level baobab genome illuminates its evolutionary trajectory and environmental adaptation
Source: Nat Commun. 2024 Oct 12;15:8833. doi: 10.1038/s41467-024-53157-w (PMC11470940; doi:10.1038/s41467-024-53157-w)
Supplement: Supplementary file 3 — Description of Additional Supplementary Files [file 41467_2024_53157_MOESM3_ESM.pdf]

Description of Additional Supplementary Files

Supplementary Data 1: Summary of the four *Adansonia digitata* genome assemblies (Ad77271a, Ad77271b, AdKB and AdOHT) and *Adansonia za* (Aza135).

Supplementary Data 2: Sequencing summary for baobab genomes.

Supplementary Data 3: Summary of structural variants in baobab genomes.

Supplementary Data 4: Comparative telomere statistics between baobab and eight other randomly selected plant species, showcasing large telomere sizes in baobab.

Supplementary Data 5: Sizes of transposable elements (bp) in baobab genomes, highlighting elevated sizes of DNA mutator elements.

Supplementary Data 6: Gene ontology terms for Ad77271a (FDR < 0.01).

Supplementary Data 7: Orthology summary for circadian, flowering, and light-related genes in baobab (Ad77271a) and five other plant species.

Supplementary Data 8: Significant gene ontology (GO) terms for 1,886 contracted genes in Ad77271a across 1,361 orthogroups (FDR < 0.01).

Supplementary Data 9: Ploidy estimation for 25 *Adansonia digitata* samples.

Supplementary Data 10: Significant gene ontology (GO) terms for African baobabs population 1 vs. 2 (FDR < 0.01).

Supplementary Data 11: Significant gene ontology (GO) terms for African baobabs population 2 vs. population 3 (FDR < 0.01).

Supplementary Data 12: Summary of Fixation Index (*Fst*) for clock genes in African baobab (*Adansonia digitata*).

Supplementary Data 13: Significant gene ontology (GO) terms for 3,212 expanded genes in Ad77271a across 878 orthogroups (FDR < 0.01).
