# Supplementary material for: Chromosome-level baobab genome illuminates its evolutionary trajectory and environmental adaptation
Source: Nat Commun. 2024 Oct 12;15:8833. doi: 10.1038/s41467-024-53157-w (PMC11470940; doi:10.1038/s41467-024-53157-w)
Supplement: Supplementary file 17 — Reporting Summary [file 41467_2024_53157_MOESM17_ESM.pdf]

nature portfolio

Corresponding author(s): Todd P. Michael

Last updated by author(s): Sep 18, 2024

Reporting Summary

Nature Portfolio wishes to improve the reproducibility of the work that we publish. This form provides structure for consistency and transparency in reporting. For further information on Nature Portfolio policies, see our Editorial Policies and the Editorial Policy Checklist.

Statistics

For all statistical analyses, confirm that the following items are present in the figure legend, table legend, main text, or Methods section.

n/a	Confirmed	
☐	☒	The exact sample size (*n*) for each experimental group/condition, given as a discrete number and unit of measurement
☒	☐	A statement on whether measurements were taken from distinct samples or whether the same sample was measured repeatedly
☐	☒	The statistical test(s) used AND whether they are one- or two-sided<br>*Only common tests should be described solely by name; describe more complex techniques in the Methods section.*
☒	☐	A description of all covariates tested
☒	☐	A description of any assumptions or corrections, such as tests of normality and adjustment for multiple comparisons
☐	☒	A full description of the statistical parameters including central tendency (e.g. means) or other basic estimates (e.g. regression coefficient) AND variation (e.g. standard deviation) or associated estimates of uncertainty (e.g. confidence intervals)
☐	☒	For null hypothesis testing, the test statistic (e.g. *F*, *t*, *r*) with confidence intervals, effect sizes, degrees of freedom and *P* value noted<br>*Give P values as exact values whenever suitable.*
☒	☐	For Bayesian analysis, information on the choice of priors and Markov chain Monte Carlo settings
☒	☐	For hierarchical and complex designs, identification of the appropriate level for tests and full reporting of outcomes
☒	☐	Estimates of effect sizes (e.g. Cohen's *d*, Pearson's *r*), indicating how they were calculated

Our web collection on statistics for biologists contains articles on many of the points above.

Software and code

Policy information about availability of computer code

Data collection	No software was utilized for sample collection
Data analysis	Flye (v2.9.2), Racon (v1.5.0), Pilon (v1.24),Juicer (v1.6.2), 3ddna (v180419), JBAT (v1.11.08) ,BUSCO (v. 5.4.3),Jellyfish (v2.2.10),GenomeScope2, nQuire,Ragtag (v2.1.0), EDTA (v1.9.6) ,Tandem Repeats Finder (v4.09),Funannotate (v1.8.2),Eggnog-mapper (v2.0.1), LoReMe (Long Read Methylation), Loreme Dorado-convert tool (v.0.3.1), Modkit (v0.1.11),OrthoFinder (v2.5.5), CAFE (v5.0),CafePlotter, CoGe,MCscan-python version, Syri (v1.6.3),Trim Galore (v0.6.6), minimap2 (v2.20), Samtools (v1.12), freebayes (v1.3.5), vcftools (v0.1.16), rtg tools (v3.12), goatools (v1.3.11), R 4.3.1

For manuscripts utilizing custom algorithms or software that are central to the research but not yet described in published literature, software must be made available to editors and reviewers. We strongly encourage code deposition in a community repository (e.g. GitHub). See the Nature Portfolio guidelines for submitting code & software for further information.

Data

Policy information about availability of data

All manuscripts must include a data availability statement. This statement should provide the following information, where applicable:

- Accession codes, unique identifiers, or web links for publicly available datasets
- A description of any restrictions on data availability
- For clinical datasets or third party data, please ensure that the statement adheres to our policy

Raw datasets for figures are in https://doi.org/10.6084/m9.figshare.26039878. Other datasets, including variants and annotations are available in our baobab

database portal at https://resources.michael.salk.edu/baobab/index.html. Additional information and raw data are available at https://zenodo.org/records/4959282, they were originally reported by Karimi et.al 2020. The raw reads for reference genome (Ad77271 a and b) have been deposited in the NCBI database under Bioproject PRJNA1022505, with specific accession numbers provided for Oxford Nanopore Technologies (ONT) (SRR26283140), Illumina Hi-C (SRR26223171), and Illumina short reads (SRR26223172). Additionally, the genomes are accessible on CoGe (https://genomevolution.org/User.pl?p=genome) under respective IDs for Ad77271a (id67797), Ad77271b (id67801), AdKB (id67798), AdOHT (id67799), Aza135 (id67800), A. grandidieri (id67794), A. gregorii (id67793), A. madagascariensis (id67790), A. perrieri (id67791), A. rubrostipa (id67795), A. suarezensis (id67796), and A. za (id67792).

Research involving human participants, their data, or biological material

Policy information about studies with human participants or human data. See also policy information about sex, gender (identity/presentation), and sexual orientation and race, ethnicity and racism.

Reporting on sex and gender	N/A
Reporting on race, ethnicity, or other socially relevant groupings	N/A
Population characteristics	N/A
Recruitment	N/A
Ethics oversight	N/A

Note that full information on the approval of the study protocol must also be provided in the manuscript.

Field-specific reporting

Please select the one below that is the best fit for your research. If you are not sure, read the appropriate sections before making your selection.

☐ Life sciences ☐ Behavioural & social sciences ☒ Ecological, evolutionary & environmental sciences

For a reference copy of the document with all sections, see nature.com/documents/nr-reporting-summary-flat.pdf

Ecological, evolutionary & environmental sciences study design

All studies must disclose on these points even when the disclosure is negative.

Study description	Our research explores the evolutionary history of the baobab tree, one of Earth's oldest non-clonal plants. Motivated by its recognition by the Food and Drug Administration (FDA) as a food ingredient and events such as the 2016 demise of the Chapman baobab, we aim to provide high-quality genomic resources and valuable evolutionary insights. The study reveals several important findings. Firstly, we determine that the African baobab (Adansonia digitata) is autotetraploid, distinguishing it from the seven other diploid species. Additionally, we observe a higher proportion of DNA transposable elements (TEs) compared to long terminal repeat retrotransposons (LTR-RTs), highlighting the unusual genomic structure of baobab. Specific Mutator transposons (TE_00000631, TE_00000845, TE_00000927, and TE_00000967) are identified in centromeric regions, suggesting their involvement in the creation or repositioning of centromeres. The study identifies two whole-genome multiplication events: one approximately 30 million years ago (MYA) and another between 3-11 MYA, resulting in up to 16 copies of genes related to flower development, chromatin/transcription, and exocytosis. We hypothesize that genes associated with UV-B light perception and chromatin regulation play a role in promoting the longevity of baobab trees. Additionally, our investigation reveals distinct baobab populations in Namibia, each linked to specific watersheds, indicating geographical barriers to gene flow and the need for targeted species conservation. These findings provide valuable data for informed breeding strategies and conservation efforts.
Research sample	The samples used in this study were selected based our previous work on baobabs (Karimi et.al 2020). Briefly; seedlings at the two true leaf stages were used for High Molecular Weight (HMW) DNA and RNA extraction, they were dark adapted for two days to deplete the polysaccharides. After two days of dark adaptation, two PI77271 seedlings were chosen for genome sequencing and named "Ad77271a" and "Ad77271b;" (seeds were obtained from GRIN- PI 77271). HMWwere also extracted for AdOHT, AdKB and Aza135. Silica-dried leaf materials were used for sequencing eight baobab species and 25 A.digitata
Sampling strategy	The sampling procedure was previously documented by Karimi et al. in 2020 and Karimi et. al 2022. Sampling was conducted across Africa to encompass diverse geographic and habitat variations. For the creation of a reference genome, seeds accessible from GRIN were chosen.
Data collection	Using next-generation sequencing technology, including Illumina and Oxford Nanopore Technologies (ONT), we conducted sequencing, assembly, and scaffolding. These efforts resulted in the development of two chromosome-scale haploid assemblies for Adansonia digitata. Additionally, we produced two long-read contig assemblies for baobabs from Namibia (AdOHT) and Sudan (AdKB), along with a single long-read contig assembly for the diploid Adansonia za (Aza135) from Madagascar. Concurrently, we utilized 25 Adansonia digitata specimens to obtain variants for population genetics analyses.
Timing and spatial scale	Samples were collected and data analysis was conducted between 2018 and 2024.
Data exclusions	No exclusion of data

Reproducibility	Raw reads are publicly available for download, and utilizing the software and options described in the article's methods section will enable the reproduction of our results
Randomization	As no clinical experiments were conducted, randomization was unnecessary
Blinding	Blinding was not feasible as the study did not involve treatment versus control groups

Did the study involve field work? ☒ Yes ☐ No

Field work, collection and transport

Field conditions	The study encompassed regions ranging from low to high altitudes in world, characterized predominantly by dry field conditions.
Location	Zimbabwe,Namibia, Botswana, Benin, Burkina Faso, Cote divoire, Tanzania, Sao Tome, South Africa, Sudan, Togo, Madagascar and Australia
Access & import/export	Access was achieved by air to the nearest airport, followed by a combination of vehicle travel and trekking, with assistance from local guides who are acknowledged in the manuscript. All necessary permissions were obtained from local authorities in each country.
Disturbance	Little to no disturbance occurred as only small leaf samples were collected

Reporting for specific materials, systems and methods

We require information from authors about some types of materials, experimental systems and methods used in many studies. Here, indicate whether each material, system or method listed is relevant to your study. If you are not sure if a list item applies to your research, read the appropriate section before selecting a response.

Materials & experimental systems

n/a	Involved in the study	
☒	☐	Antibodies
☒	☐	Eukaryotic cell lines
☒	☐	Palaeontology and archaeology
☒	☐	Animals and other organisms
☒	☐	Clinical data
☒	☐	Dual use research of concern
☐	☒	Plants

Methods

n/a	Involved in the study	
☒	☐	ChIP-seq
☒	☐	Flow cytometry
☒	☐	MRI-based neuroimaging

Dual use research of concern

Policy information about dual use research of concern

Hazards

Could the accidental, deliberate or reckless misuse of agents or technologies generated in the work, or the application of information presented in the manuscript, pose a threat to:

No	Yes	
☒	☐	Public health
☒	☐	National security
☒	☐	Crops and/or livestock
☒	☐	Ecosystems
☒	☐	Any other significant area

Experiments of concern

Does the work involve any of these experiments of concern:

No	Yes	
☒	☐	Demonstrate how to render a vaccine ineffective
☒	☐	Confer resistance to therapeutically useful antibiotics or antiviral agents
☒	☐	Enhance the virulence of a pathogen or render a nonpathogen virulent
☒	☐	Increase transmissibility of a pathogen
☒	☐	Alter the host range of a pathogen
☒	☐	Enable evasion of diagnostic/detection modalities
☒	☐	Enable the weaponization of a biological agent or toxin
☒	☐	Any other potentially harmful combination of experiments and agents

Plants

Seed stocks	Seeds for reference genome samples were acquired from the USDA Germplasm Information Resource Network (GRIN), originating from three trees cultivated at the USDA-Agriculture Research Service, Subtropical Horticulture Research Station in Miami, FL, USA, identified by the accession number PI 77271
Novel plant genotypes	No novel genotypes were developed in this study
Authentication	Seeds were freely provided by GRIN
